# Psychometric Properties and Factor Structures of Chinese Smartphone Addiction Inventory: Test of Two Models

**DOI:** 10.3389/fpsyg.2018.01411

**Published:** 2018-08-06

**Authors:** Hsin-Yi Wang, Leif Sigerson, Hongyan Jiang, Cecilia Cheng

**Affiliations:** ^1^Department of Psychology, The University of Hong Kong, Pokfulam, Hong Kong; ^2^School of Management, China University of Mining and Technology, Xuzhou, China

**Keywords:** smartphone addiction, technology addiction, mobile phone, factor analysis, scale validation, psychometric properties

## Abstract

There has been a growing concern of excessive smartphone use that interferes with people’s daily functioning, most notably among youngsters. The Smartphone Addiction Inventory (SPAI) was constructed to assess this type of information technology addiction. Although the SPAI was developed in a Taiwanese adolescent sample, this measure has not been validated on Chinese youngsters in other regions. Moreover, the initial evidence yielded a four-factor structure, but recent findings obtained an alternative five-factor structure. As no studies have systematically compared these two factor structures, which of the models fits the data better remained unknown. This study aimed to evaluate the empirical validity of both the four- and five-factor structures of the SPAI in a sample of university students from Mainland China (*n* = 463). Four psychometric properties of the SPAI were examined. First, the structural validity of both factor models was evaluated with confirmatory factor analysis. Satisfactory fit was found for both the five-factor model (RMSEA = 0.06, SRMR = 0.05, CFI = 0.99, TLI = 0.99) and the four-factor model (RMSEA = 0.07, SRMR = 0.06, CFI = 0.98, TLI = 0.98), but the five-factor model demonstrated an overall better model fit. Second, the five-factor model yielded good internal consistencies (all Cronbach α’s > 0.70). Third, concurrent validity of the SPAI was supported by its moderately strong correlations with four widely adopted criterion variables (i.e., loneliness, social anxiety, depression, and impulsivity). Lastly, the convergent validity of the SPAI was demonstrated by its strong, positive correlation with a popular, validated measure of Internet addiction. This study is the first to demonstrate the validity of the newly proposed five-factor model of the SPAI in a sample of Mainland Chinese youngsters.

## Introduction

The prevalence of smartphone use has rapidly increased across the globe over the past decade (Poushter, 2016). As of 2015, approximately 73% of adolescents in the United States and 60% of those in Mainland China have access to smartphones (Lenhart et al., 2015; CNNIC, 2016). Nowadays, many adolescent users consider smartphones as their primary tool to browse the Internet due to its accessibility and affordability (Lenhart et al., 2015). Despite the convenience and efficiency brought by this mobile device, recent research has demonstrated that excessive use of smartphone can be psychologically detrimental to adolescent users (e.g., Davey and Davey, 2014).

Smartphone addiction is a term which describes the pathological use of the mobile device that severely disturbs users’ daily life functioning (Lin et al., 2014), and this addictive use encompasses a wide range of activities such as Internet gaming and online social networking (Billieux, 2012). Although researchers and practitioners have yet to reach consensus on the formal diagnostic criteria of smartphone addiction and other types of behavioral addiction (Kardefelt-Winther et al., 2017), some scholars have conceptualized smartphone addiction as a type of behavioral addiction that is centered around human–smartphone interactions (Lin et al., 2016). Several recurring symptoms of this addiction have also been proposed, including compulsion to engage in smartphone-related activities despite awareness of aversive consequences (Lee Y. K. et al., 2014; Lin et al., 2017), disturbance in daily-life functioning such as time management issues and sleep interferences (Lin et al., 2015; Liu et al., 2017), increased amount of time spent on smartphone (Lee H. et al., 2014) and withdrawal symptoms following abstinence from smartphone use (Clayton et al., 2015).

In addition to the core symptoms, several psychological factors have also been identified to be associated with smartphone addiction, most notably loneliness, social anxiety, depression, and impulsivity. For lonely smartphone users who are dissatisfied with their offline social relations, various instant communication applications available on smartphone (e.g., direct messaging, online social networking, and video chat) allow these users to be constantly connected with other members from their online social networks (Kim, 2018). Also, these communication applications provide a less-pressuring alternative to maintain social relations for individuals who feel anxious in face-to-face social interactions (Enez Darcin et al., 2016). However, over-reliance on smartphone for social connections can result in unregulated usage of this device for individuals who experience loneliness and social anxiety (e.g., Bian and Leung, 2015; Enez Darcin et al., 2016).

Smartphone users who experience depressive symptoms may similarly utilize their mobile devices as a coping strategy to mitigate these unpleasant symptoms (Kim et al., 2015). When these users experience unpleasant mood, using smartphone is an immediate coping strategy to deflect attention from their adverse feelings through engaging in various online entertainment services, avoiding engaging in real-life activities such as communicating with others, or both (Elhai et al., 2017). Nonetheless, reliance on smartphone to relieve depressive symptoms without directly handling the source of depression can increase smartphone usage and eventually elicit smartphone addiction (Kim et al., 2015).

Apart from psychological problems, personality factors such as impulsivity are also associated with smartphone addiction (Kim et al., 2016). Individuals with higher impulsivity levels are more likely to encounter difficulties in concentration due to irrelevant or unwanted thoughts (Billieux, 2012). Meanwhile, the array of activities instantly available on smartphone can alleviate their boredom or frustrations generated from an inability to concentrate while accomplishing tasks (Roberts et al., 2015). Thus, impulsivity has been consistently identified as a major personality risk factor of smartphone addiction (e.g., Roberts et al., 2015; Contractor et al., 2017). In addition, researchers often emphasized the necessity to scrutinize multiple facets of impulsivity (Stanford et al., 2009) because different facets are found to have distinct associations with Internet addiction. Specifically, studies have indicated that Internet addiction has stronger associations with motor impulsivity (i.e., acting without careful thinking) and attentional impulsivity (i.e., making quick and abrupt decisions), but weaker associations with non-planning impulsivity (i.e., lack of future-oriented planning and forethoughts) (e.g., Cao et al., 2007; Zhou et al., 2010).

It is noteworthy that individuals with smartphone addiction tend to engage in similar addictive activities as those having Internet addiction (Kwon et al., 2013a; Van Deursen et al., 2015). For instance, Internet addiction is positively associated with not only smartphone addiction but also general smartphone usage (Ben-Yehuda et al., 2016). In addition, smartphone addiction and Internet addiction share some common symptoms such as withdrawal and functional impairment (Lin et al., 2014) as well as similar psychological risk factors such as social anxiety (e.g., Billieux, 2012; Choi et al., 2015).

Despite sharing several common symptoms and risk factors, smartphone addiction and Internet addiction are found to be conceptually distinct (Sigerson et al., 2017). Compared to other Internet devices such as personal computers, the portability and availability of smartphones allow users to engage in addictive activities under a plethora of circumstances that are not possible for other information technology devices (e.g., Choi et al., 2015; Liu et al., 2016). For instance, distracting smartphone use in a classroom environment is positively associated with the severity of smartphone addiction because students can still use smartphones but not computers while attending classes (Gökçearslan et al., 2016). Moreover, improper smartphone use among pedestrians has now become a public health concern, as individuals with smartphone addiction report higher numbers of unintentional pedestrian injuries than smartphone users without this problem (Tao et al., 2015). These unique characteristics of smartphone addiction signify the necessity to conceptually differentiate smartphone addiction from other types of information technology addiction.

Although the prevalence and adverse influences of smartphone addiction have alerted scholars and policymakers in Mainland China, there is a lack of standardized instruments to assess smartphone addiction among Chinese adolescents. In the literature, the estimated prevalence rate of smartphone addiction among Chinese adolescents ranged from 21 to 38% (e.g., Tao et al., 2015; Wang and Zhang, 2015; Long et al., 2016). This large between-study discrepancy in prevalence rates may be attributable to the adoption of diverse assessment tools of smartphone addiction. Previous research in Mainland China has commonly adopted instruments that are not specifically tailored for smartphones, such as Mobile Phone Addiction Inventory (MPAI; Huang et al., 2014) and Problematic Cellular Phone Use Questionnaire (PCPUQ; Yen et al., 2009). The major problems of these instruments rest on their primary focus on the communication functions of mobile phones such as text messaging and voice calling, but scant emphasis has been placed on online features of smartphone such as website browsing and online gaming. Even for assessment tools that emphasize the online features of smartphone, there is a lack of consensus about the official diagnostic criteria of smartphone addiction. As a result, different assessment tools focus on distinct aspects of smartphone addiction. For example, the Smartphone Addiction Scale originally developed in South Korea (Kwon et al., 2013b) includes several items assessing individuals’ positive anticipation and gratification of smartphone use, but such items are absent in other instruments such as the Smartphone Addiction Inventory (SPAI; Lin et al., 2014). Thus, the distinct conceptualizations and operationalization of smartphone addiction across various assessment tools may also contribute to the inconsistent prevalence rates reported in previous studies.

To the best of our knowledge, the SPAI (Lin et al., 2014) is currently the only measure designed to specifically assess smartphone addiction among Chinese users. However, the SPAI has only been validated with Chinese adolescents in Taiwan, and has yet to be empirically validated with those from other regions. Although the Chinese in Taiwan and Mainland China share considerable degrees of ethnical similarity, the differences in information technology infrastructure in these two regions may have substantial influences on smartphone usage of their residents. From the year of 2011, various mobile payment services have enabled consumers in Mainland China to pay for a wide range of services and products through their smartphones (Lu, 2018). In contrast, cash still remains the dominant payment option for offline purchases in Taiwan. These life-style differences may have significant impacts on the way adolescents perceive their smartphones and their usage, as the majority of adolescents and young adults in Mainland China consider smartphones as their default payment option (Lu, 2018). Thus, it is imperative to further examine the validity of SPAI with youngsters in Mainland China.

The SPAI is a self-administered instrument designed to assess the level of smartphone addiction among Taiwanese university students. The scale was developed based on the conceptualization that smartphones provide not only communicating services but also multiple online functions. Thus, the Revised Chen Internet Addiction Scale (CIAS-R; Chen et al., 2003) was chosen as the basis for constructing the SPAI items. To modify the original items, the term “Internet use” in the CIAS-R was replaced with “smartphone use” in the SPAI. For example, “I fail to control the impulse to use Internet” was modified to “I fail to control the impulse to use smartphone,” and “My life would be joyless hadn’t there been Internet” was modified to “My life would be joyless hadn’t there been smartphone.”

The initial validation of the SPAI yielded four factors representing the core components of smartphone addiction: (a) “compulsive behavior” of using smartphone that cannot be controlled by users despite the experience of undesirable outcomes, (b) “functional impairment” such as sleep disturbances and time management issues associated with smartphone use, (c) “withdrawal” symptoms including feelings of restlessness and unease following abstinence from smartphone use, and (d) “tolerance” symptoms which refer to the increasing amount of time spent on smartphone in order to attain the same level of need satisfaction.

In addition to the initial validation (Lin et al., 2014), the SPAI has been translated and further validated in samples from two other nations. The Brazilian version of the SPAI (SPAI-BR; Khoury et al., 2017) has been found to be a valid tool for assessing smartphone addiction among university students in Brazil. However, the Italian version of the SPAI (SPAI-I; Pavia et al., 2016) found unsatisfactory results with the four-factor structure proposed in the initial study by Lin et al. (2014), and further analysis revealed a five-factor model with good structural validity. With two items having low factor loadings removed from the scale, the revised factorial structure comprises five factors that categorized smartphone addiction symptoms in a distinct manner, including time spent on smartphone, compulsivity to use smartphone, daily life interference, craving for smartphone use, and sleep interference. Pavia et al. (2016) proposed that the discrepancy between the two factor models may stem from the cultural differences between Italian and Taiwanese participants, but the discrepancy may also be due to a lack of consensual definition concerning the symptomology of smartphone addiction. Hence, a direct comparison of the four-factor and five-factor models within the same Chinese sample will provide further insights into the theoretical significance of the revised five-factor structure.

The present study aimed to evaluate whether the SPAI is an appropriate instrument for assessing smartphone addiction among adolescents in Mainland China. To realize this aim, we first tested the structural validity and reliability of the four-factor SPAI model structure initially obtained by Lin et al. (2014), as well as the five-factor SPAI-I model structure identified by Pavia et al. (2016). Then we examined the convergent validity of the SPAI with another major type of information technology addiction, Internet addiction. Lastly, we investigated the concurrent validity of the SPAI with four major psychological risk factors of smartphone addiction, including loneliness, social anxiety, depression, and impulsivity (including three facets: motor, non-planning, and attentional).

## Materials and Methods

### Participants

Five hundred Chinese university students were initially recruited for this study, but 33 who failed to complete the study and four non-smartphone users were excluded. The final sample of 463 participants consisted of 78% men, with an average age of 18.75 years (*SD* = 0.99, range = 18–20).

### Procedures

Participants were recruited from a university in an urban region of Mainland China. Instructors of several undergraduate courses were contacted for recruiting their students as participants. After receiving approval from these instructors, data collection was conducted in a classroom setting. Before the study began, all of the participants were told that course credits would be available for taking part in this study and informed consent was obtained. A set of questionnaires was distributed to participants for their completion. Participants were thanked and debriefed after they had filled in the questionnaires, and course credits were then assigned.

### Measures

#### Smartphone Addiction Inventory

The Smartphone Addiction Inventory (SPAI; Lin et al., 2014) was adopted to measure smartphone addiction. This scale included 26 items that were originally categorized into four dimensions: functional impairment (8 items), withdrawal (6 items), compulsive behavior (9 items), and tolerance (3 items). Participants were asked to rate these items on a 4-point Likert scale, ranging from 1 (*strongly disagree*) to 4 (*strongly agree*). The SPAI displayed excellent reliability in the present sample (see the “Results” section for details). Both English and Chinese versions of the SPAI are listed in **Appendix 1**.

#### Revised Chen Internet Addiction Scale

The Revised Chen Internet Addiction Scale (CIAS-R; Chen et al., 2003) was selected to assess Internet addiction because it was constructed specifically for Chinese populations. Respondents answered 26 items that comprised five dimensions: symptoms of compulsive use (5 items), withdrawal (5 items), tolerance (4 items), problems in interpersonal relations (7 items), as well as health and time management (5 items). Each item was measured on a 4-point Likert scale, ranging from 1 (*does not match my experience*) to 4 (*definitely matches my experience*). This scale was further validated in a sample of Chinese adolescents (Mak et al., 2014). The CIAS-R had excellent reliability in our sample (Cronbach’s α = 0.95).

#### Short-Form UCLA Loneliness Scale

The short form of the UCLA Loneliness Scale (ULS-8; Hays and DiMatteo, 1987) was chosen because it was the most commonly adopted measure of perceived loneliness. This unidimensional measure evaluates the core experiences of loneliness in 8 items. Respondents rated these items on a 4-point Likert scale (1 = *never* to 4 = *always*). This measure was validated with a sample of Chinese adolescents in Taiwan (Wu and Yao, 2008). The ULS-8 was a reliable measure in this study (Cronbach’s α = 0.76).

#### Short-Form Social Anxiety Interaction Scale

The short form of the Social Anxiety Interaction Scale (SIAS-6; Peters et al., 2012) was selected to measure social anxiety due to its popularity in use among social anxiety researchers. This scale adopts a unidimensional measurement approach. Respondents were instructed to rate 6 items measuring their level of anxiety regarding initiating and maintaining social interactions in a 5-point Likert scale, ranging from 1 (*not at all characteristic or true of me*) to 5 (*extremely characteristic or true of me*). The full-length Chinese version of SIAS has been previously validated (Yang, 1997). Good reliability of this scale was found with the current sample (Cronbach’s α = 0.86).

#### Short-Form Center for Epidemiological Studies Depression Scale

The short form of the Center for Epidemiological Studies Depression Scale (CES-D 10; Cole et al., 2004) was employed to assess depression because this is also a very popular measure of depressive symptoms. This instrument is developed as a unidimensional measure. Respondents completed 10 items measuring the frequency and duration of a set of depressive symptoms. Each item was measured in a 4-point Likert scale, ranging from 1 (*rarely or none of the time*) to 4 (*most or all of the time*). The CES-D 10 was validated in Chinese samples (Cheng and Chan, 2005; Cheung et al., 2007). There was good reliability of this scale in the present study (Cronbach’s α = 0.76).

#### Short-Form Barratt Impulsiveness Scale

The short form of the Barratt Impulsiveness Scale (BIS-15; Spinella, 2007) was adopted to measure impulsivity. This commonly adopted measure assessed various behavioral and psychological indicators of impulsivity. Respondents rated 15 items in a 4-point Likert scale (1 = *rarely/never* to 4 = *almost/always*). The BIS-15 is comprised of three subscales: motor impulsivity (5 items), non-planning impulsivity (5 items) and attentional impulsivity (5 items). The full-length BIS had been previously validated in a sample of Chinese adolescents (Wan et al., 2016). This scale displayed good reliability in this study (Cronbach’s α = 0.72).

## Results

### Structural Validity and Reliability

The structural validity of the four-factor SPAI model and the five-factor SPAI-I model were examined with confirmatory factor analysis, which was performed using the Lavaan package version 5.20 (Rosseel, 2012) in R version 3.4.1. As the scale items had four response options, diagonally weighted least squares (DWLS) was used to estimate the model (Rhemtulla et al., 2012). In order to evaluate the extent of match between the proposed models and the current data, four well-established goodness-of-fit indices were selected: Root Mean Square Error of Approximation (RMSEA), Standardized Root Mean Square Residual (SRMR), Comparative Fit Index (CFI), and Tucker-Lewis Index (TLI). To evaluate the overall model fit, we followed the conventional criteria (Hu and Bentler, 1999): RMSEA < 0.08, SRMR < 0.08, CFI > 0.90, and TLI > 0.90.

**FIGURE 1:**
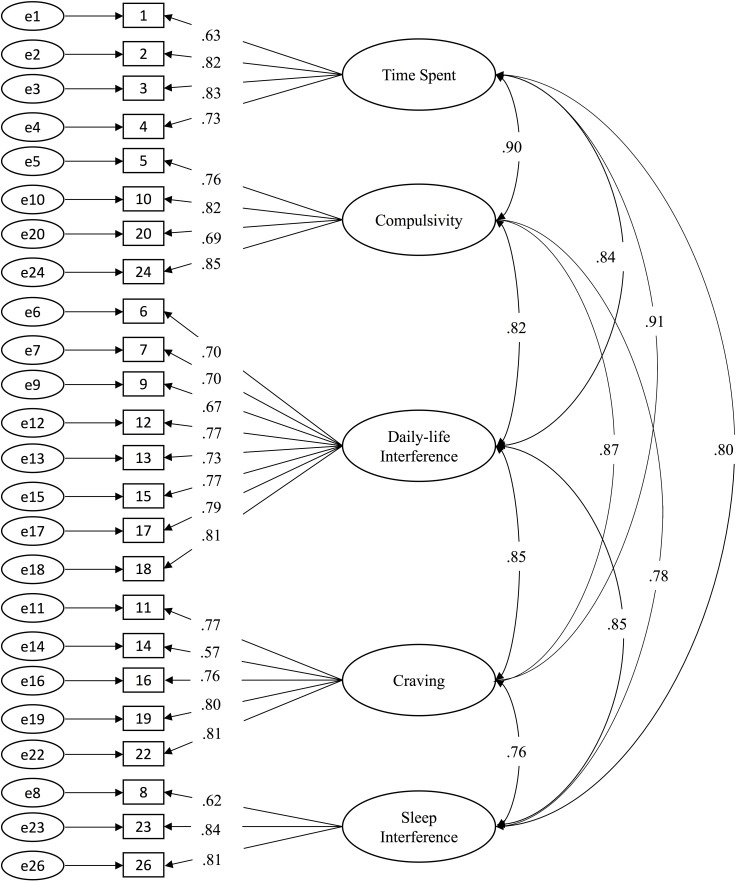
The five-factor SPAI-I model and its standardized factor loadings.

The four-factor SPAI model had model fit indices of RMSEA = 0.07, SRMR = 0.06, CFI = 0.98, TLI = 0.98. The five factor SPAI-I model had consistently better model fit indices: RMSEA = 0.06, SRMR = 0.05, CFI = 0.99, TLI = 0.99. All of the factor loadings in both models were statistically significant with values larger than 0.50. Overall, these findings showed that the five-factor model displayed a better fit than the four-factor model, and thus all the subsequent analyses were performed based on the five-factor SPAI-I model. The SPAI-I model is depicted in **Figure 1**.

The reliability of the five-factor SPAI-I model was assessed with Cronbach’s alpha. The overall scale displayed good reliability (Cronbach’s α = 0.94), and so did each of the subscales (i.e., Cronbach’s α for time spent: 0.78, compulsivity: 0.80, daily-life interference: 0.87, craving: 0.82, and sleep interference: 0.73). These findings showed that the SPAI-I and all its subscales were reliable.

### Convergent Validity Analysis

To evaluate the convergent validity of the five-factor SPAI-I model, we examined Pearson zero-order correlations between the SPAI-I and CIAS-R. As shown in **Table 1**, there was a strong positive association between the SPAI-I composite score and the CIAS-R score (*r* = 0.75, *p* < 0.001). In addition, each of the SPAI-I subscales was positively associated with the CIAS-R score (*r* ranges from 0.63 to 0.71). All these results demonstrated convergent validity of the SPAI-I.

**Table 1:** Pearson zero-order correlation analyses for the SPAI-I and its five subscales.

	SPAI-I sum score	Time spent	Compulsivity	Daily-life interference	Craving	Sleep interference
**Convergent validity**					
Internet addiction	0.75^∗∗^	0.64^∗∗^	0.65^∗∗^	0.71^∗∗^	0.63^∗∗^	0.64^∗∗^
**Concurrent validity**					
Loneliness	0.34^∗∗^	0.29^∗∗^	0.29^∗∗^	0.33^∗∗^	0.25^∗∗^	0.31^∗∗^
Social anxiety	0.36^∗∗^	0.33^∗∗^	0.35^∗∗^	0.33^∗∗^	0.31^∗∗^	0.30^∗∗^
Depression	0.32^∗∗^	0.27^∗∗^	0.30^∗∗^	0.28^∗∗^	0.25^∗∗^	0.29^∗∗^
Impulsivity (total)	0.43^∗∗^	0.36^∗∗^	0.37^∗∗^	0.38^∗∗^	0.35^∗∗^	0.39^∗∗^
Motor impulsivity	0.34^∗∗^	0.29^∗∗^	0.31^∗∗^	0.32^∗∗^	0.26^∗∗^	0.31^∗∗^
Non-planning impulsivity	0.20^∗∗^	0.14	0.12	0.18^∗∗^	0.15^∗∗^	0.15^∗∗^
Attentional impulsivity	0.38^∗∗^	0.34^∗∗^	0.36^∗∗^	0.30^∗∗^	0.34^∗∗^	0.38^∗∗^


^∗∗^p < 0.001 (critical **P-value after Bonferroni correction: 0.05/48 = 0.001).

### Concurrent Validity Analysis

To examine the concurrent validity of the SPAI, we again performed correlational analysis of the SPAI-I with its four major psychological risk factors. To control for the potential Type I errors related to multiple comparisons, Bonferroni correction was applied to the present analysis (Curtin and Schulz, 1998). As shown in **Table 1**, the associations of SPAI-I with loneliness, social anxiety, depression, and impulsivity were all positive and moderately strong (*r*’s ranged from 0.32 to 0.43). Similar associations were found between all five SPAI-I subscales and these criterion variables (*r*’s ranged from 0.25 to 0.39). All of these associations were significant (*p*’s < 0.001). Both sets of results were in line with our predictions derived from the existing literature, thus providing evidence for concurrent validity of the SPAI-I.

To further scrutinize the multifaceted construct of impulsivity, an additional analysis was performed for the three subscales of the impulsivity measure. The findings revealed positive associations of SPAI-I with motor, non-planning and attentional impulsivity (*r*’s ranged from 0.20 to 0.38). Although both motor and attentional impulsivity were positively associated with all five SPAI-I subscales (*r*’s ranged from 0.26 to 0.38), there were no significant associations between non-planning impulsivity and two of the SPAI-I subscales (*r*’s ranged from 0.12 to 0.18).

## Discussion

The present study aimed to evaluate the psychometric properties of the SPAI in a sample of Mainland Chinese adolescents. We found satisfactory model fit for the original four-factor SPAI model, as well as the more recently found five-factor SPAI-I model. However, the five-factor model is deemed as more fitting for assessing smartphone addiction based on four indices of model fit. Similar to previous validation of this model (Pavia et al., 2016), reliability is excellent for this SPAI-I model. The present results also support the concurrent validity of the SPAI-I with four major psychological risk factors of smartphone addiction. Specifically, there are robust positive associations of SPAI-I with loneliness, social anxiety, depression, and impulsivity. The magnitude of these associations is comparable to previous empirical evidence (e.g., Bian and Leung, 2015; Kim et al., 2016; Elhai et al., 2017). In addition, our analysis yielded distinct associations among SPAI-I with three facets of impulsivity. Specifically, all five SPAI-I subscales were positively associated with both motor and attentional impulsivity. However, two of the SPAI-I subscales were not directly associated with non-planning impulsivity. The current results also corroborated with past findings concerning the three facets of impulsivity (e.g., Cao et al., 2007; Zhou et al., 2010).

The present analysis also demonstrates the convergent validity of SPAI-I by revealing its strong positive association with Internet addiction. Although the strength of this association does not exceed the suggested criteria for conceptual overlap between the two constructs (Kline, 2005), the association obtained in our study is stronger than those derived from previous studies using other measures to examine the association between smartphone addiction and Internet addiction (Choi et al., 2015; Pavia et al., 2016; Sigerson et al., 2017). This discrepancy with previous findings may occur as the SPAI is developed based on a validated measure of Internet addiction (i.e., the CIAS-R), and thus the items of these instruments may have similar wordings.

### Research and Theoretical Implications

The present findings have some implications for future research. As valid and reliable measures are essential for examining the phenomenon of smartphone addiction and designing intervention programs, this study contributes to the literature by validating the SPAI in a Mainland Chinese sample. In addition to previous validation studies in Brazil and Italy (Pavia et al., 2016; Khoury et al., 2017), our findings provide the first validational evidence for the five-factor SPAI-I model for researchers to assess smartphone addiction in an Asian adolescent sample. It is noteworthy that although the four-factor SPAI model was initially developed with a sample of adolescents in Taiwan, the five-factor SPAI-I model is more fitting for assessing smartphone addiction among Mainland Chinese adolescents. To our knowledge, this study is also the first to provide comprehensive evidence of SPAI-I’s concurrent and convergent validity in a Chinese sample. Future research should build on the current findings to further clarify the influences of these aforementioned psychological factors on the development of smartphone addiction.

In addition to validating the psychometric properties of the four-factor and the five-factor models, our comparison between the two factorial structures also advance the conceptual understanding of smartphone addiction. Our findings highlight the importance to incorporate unique characteristics of smartphones into the conceptualization of smartphone addiction. Specifically, sleep interferences resulting from smartphone use should be distinguished from other aspects of daily-life interferences. To address these distinctions between the two factorial structures, Pavia et al. (2016) proposed that the discrepancy may be attributable to cross-sample distinctions such as gender ratio and cultural context, or this structural difference may stem from inconsistent understanding of smartphone addiction among scholars. As the present Mainland Chinese sample and the Taiwanese sample involved in the initial development of the SPAI (Lin et al., 2014) are largely similar in gender composition and ethnicity, the less optimal model fit of the four-factor SPAI model may indicate a lack of consensus on the conceptualization of smartphone addiction.

One of the major difference between the two models rests on the five-factor SPAI-I model’s discernment of three items assessing sleep-related issues from other items tapping daily-life interferences. Although sleep interference is deemed a core symptom for both smartphone addiction and Internet addiction (see Cain and Gradisar, 2010 for a review), the nature of these interferences differs vastly across devices. Similar to other aspects of daily-life interferences such as time management, the proposed mechanisms for the association between sleep-related issues and Internet addiction have primarily focused on compulsive and excessive Internet use that depletes users’ sleep time (Chen and Gau, 2016).

As recent evidence has demonstrated, sleep interferences resulting from bedtime smartphone use can explain the findings beyond the usage of other information technology devices such as personal computers and tablets (Lanaj et al., 2014). Hence, there are specific characteristics of smartphone that may induce sleep-related problems, in addition to general sleep depletion due to Internet use. Compared with personal computers, smartphones are portable that allow individuals to use this device immediately before they attempt to fall asleep (Exelmans and Van den Bulck, 2016), and approximately 70% of users keep their smartphone by their bedside before sleep (Gibbs, 2012). This prevalent pattern of smartphone use is particularly problematic, as the notification features of smartphone (e.g., ring tones and vibrations) can severely disturb user’s sleep pattern (e.g., Lanaj et al., 2014; Ahn and Kim, 2015). In addition, recent evidence has shown that exposure to screen light, specifically blue light, of smartphone during bedtime can also reduce sleep duration and quality (Christensen et al., 2016). Scholars have also cautioned the severity of this issue among adolescents, as most younger users primarily access their smartphones during night time due to various constraints that occur during day time, such as school regulations (Liu et al., 2017). Thus, our findings imply that sleep interference as a symptom of smartphone addiction should not be merely considered as a cognate problem of other facets of daily-life interferences; rather, such sleep interference is unique to smartphone addiction.

### Research Caveats

The present study has several caveats that call for further research attention. Specifically, our sample has a higher ratio of male participants, but previous studies have shown female (vs. male) smartphone users having higher risks of developing smartphone addiction (e.g., Choi et al., 2015; Demirci et al., 2015). Yet, the gender composition of the present sample is similar to that of the sample recruited for the initial development of the SPAI (Lin et al., 2014), thus allowing an adequate basis for conceptual comparisons between the four-factor and the five-factor models. Hence, researchers should not generalize the current findings to female Chinese adolescents without caution. Future studies should address this issue by recruiting more gender-balanced samples in order to examine whether the psychometric properties of the SPAI are similar for both male and female adolescents.

It is also noteworthy that this study recruited participants from a university located in an urban region of Mainland China. This urban adolescent sample enables us to study the psychometric properties of the SPAI among active smartphone users. However, this sampling strategy may potentially limit the generalizability of our findings to Chinese adolescents from less developed or rural regions. Although the number of adolescents in rural areas who use smartphones to assess the Internet has gradually increased, this group still only accounts for a low percentage (28%) of adolescent smartphone users (CNNIC, 2016). Moreover, recent evidence has documented differences in attitude toward smartphone between users in urban and rural areas (Hong, 2016). Most notably, adolescents in urban areas of Mainland China are more likely to perceive smartphone as a functional tool for communication and social networking (Lu, 2014), whereas those in rural areas tend to perceive smartphone as a symbol representing social status, and the latter type of smartphone perception has been found to be associated with lower levels of life satisfaction (Xie et al., 2016). Thus, future researchers should further examine the psychometric properties of SPAI in samples from less developed regions of Mainland China for investigating whether the SPAI is also valid among adolescents in these less studied regions.

Another caveat of the present study stems from the fundamental item development process of the SPAI. Both the CIAS-R (Chen et al., 2003) and the SPAI (Lin et al., 2014) adopted the diagnostic criteria of substance addiction stated in the Fourth Edition of the Diagnostic and Statistical Manual of Mental Disorders (American Psychiatric Association, 2000). This operationalization of smartphone addiction is conceptually similar to the component model of addiction, which postulates that similar to substance addiction, addictive behaviors such as smartphone use consist of several core components, including salience, withdrawal, tolerance, and relapse (Griffiths, 2005). However, this conceptualization of smartphone addiction and other behavioral addictions has been queried by some scholars and practitioners (e.g., Billieux et al., 2015; Kardefelt-Winther et al., 2017), who advocated the expansion of the scope of research beyond the conventional diagnostic criteria of substance addiction. As such theoretical advancement has yet to be made, we primarily analyzed and interpreted the present validation evidence in light of the theoretical framework of the widely adopted component model. Future researchers may further explore and compare different assessment tools and methods after new theoretical perspectives on smartphone addiction have been formulated and widely accepted by scholars and practitioners.

Finally, researchers should also pay attention to the generalizability of our findings of convergent validity to other measures. In the present study, we analyzed the direct association between smartphone addiction assessed by the SPAI and Internet addiction assessed by the CIAS-R. As the items of the SPAI are derived from those of the CIAS-R, the resemblance of item wording between these two instruments may inflate the strength of their association. For instance, the effect size of the present finding (*r* = 0.75) is considerably higher than those of previous studies (*r*’s ranged from 0.21 to 0.51) using instruments of smartphone addiction and Internet addiction that are structurally distinct (e.g., Mok et al., 2014; Choi et al., 2015; Sigerson et al., 2017). Nevertheless, this potentially inflated strength of association has also been obtained in past research using structurally similar instruments for assessing smartphone addiction and Internet addiction. For instance, the Smartphone Addiction Scale (SAS; Kwon et al., 2013b) is modified from the Korean Internet Addiction Scale (K-Scale; Kim et al., 2008), and the direct association between these two instruments are comparable to that obtained in our study. To further scrutinize these between-study discrepancies in effect size, we encourage researchers to test the replicability of the present findings using structurally distinct measures.

## Conclusion

Although the detrimental effects of smartphone addiction have been well-documented, the lack of standardized measures potentially limits the scope of relevant research. The present study contributes to the literature by examining the validity and reliability of the SPAI among adolescents in Mainland China. By comparing the two factorial structures of this instrument, our analysis indicates that the five-factor SPAI-I model is a more fitting measure to assess smartphone addiction for Chinese young smartphone users in this region. The new findings also shed light on the conceptualization of smartphone addiction, indicating that the symptom of sleep interference and other symptoms of daily-life interferences should not be regarded as cognate constructs. Future endeavors are needed to further examine the psychometric properties of SPAI among different subgroups of smartphone users.

## Ethics Statement

This study was conducted in accordance with the recommendations of ethics committee of the University of Hong Kong. All subjects gave written informed consent in accordance with the Declaration of Helsinki. The research protocol was approved by the ethics committee of the University of Hong Kong.

## Availability of Data

All the data used for the statistical analysis will be made available upon request from other researchers.

## Author Contributions

CC and HJ designed the study. HJ coordinated the data collection process. H-YW and LS performed the statistical analysis. H-YW completed the first draft of the manuscript. All authors contributed to the editing and revision of the manuscript.

## Conflict of Interest Statement

The authors declare that the research was conducted in the absence of any commercial or financial relationships that could be construed as a potential conflict of interest.

## Appendix 1

**Table:** Items of Smartphone Addiction Inventory (English).

Items	
(1)	I was told more than once that I spent too much time on smartphone.
(2)	I feel uneasy once I stop smartphone for a certain period of time.
(3)	I find that I have been hooking on smartphone longer and longer.
(4)	I feel restless and irritable when the smartphone is unavailable.
(5)	I feel very vigorous upon smartphone use regardless of the fatigues experienced.
(6)	I use smartphone for a longer period of time and spend more money than I had intended.
(7)	Although using smartphone has brought negative effects on my interpersonal relationships, the amount of time spent on Internet remains unreduced.
(8)	I have slept less than 4 h due to using smartphone more than once.
(9)	I have increased substantial amount of time using smartphone per week in recent 3 months.
(10)	I feel distressed or down once I cease using smartphone for a certain period of time.
(11)	I fail to control the impulse to use smartphone.
(12)	I find myself indulged on the smartphone at the cost of hanging out with friends.
(13)	I feel aches and soreness in the back or eye discomforts due to excessive smartphone use.
(14)	The idea of using smartphone comes as the first thought on mind when wake up each morning.
(15)	To use smartphone has exercised certain negative effects on my schoolwork or job performance.
(16)	I feel missing something after stopping smartphone for a certain period of time.
(17)	My interaction with family members is decreased on account of smartphone use.
(18)	My recreational activities are reduced due to smartphone use.
(19)	I feel the urge to use my smartphone again right after I stopped using it.
(20)	My life would be joyless hadn’t there been smartphone.
(21)	Surfing the smartphone has exercised negative effects on my physical health. For example, viewing smartphone when crossing the street; fumbling with one’s smartphone while driving or waiting, and resulted in danger.
(22)	I try to spend less time on smartphone, but the efforts were in vain.
(23)	I make it a habit to use smartphone and the sleep quality and total sleep time decreased.
(24)	I need to spend an increasing amount of time on smartphone to achieve same satisfaction as before.
(25)	I can not have meal without smartphone use.
(26)	I feel tired on daytime due to late-night use of smartphone.

**Table:** Items of Smartphone Addiction Inventory (Chinese).
